# Effect of Adherence to a Mediterranean Diet and Olive Oil Intake during Pregnancy on Risk of Small for Gestational Age Infants

**DOI:** 10.3390/nu10091234

**Published:** 2018-09-05

**Authors:** Juan Miguel Martínez-Galiano, Rocío Olmedo-Requena, Rocío Barrios-Rodríguez, Carmen Amezcua-Prieto, Aurora Bueno-Cavanillas, Inmaculada Salcedo-Bellido, Jose J. Jimenez-Moleon, Miguel Delgado-Rodríguez

**Affiliations:** 1 Department of Nursing, University of Jaén, Building B3 Office 266, Campus de Las Lagunillas s/n, 23071 Jaén, Spain; 2 Consortium for Biomedical Research in Epidemiology and Public Health (CIBERESP), 28029 Madrid, Spain; rocioolmedo@ugr.es (R.O.-R.); rbarrios@ugr.es (R.B.-R.); carmezcua@ugr.es (C.A.-P.); abueno@ugr.es (A.B.-C.); isalcedo@ugr.es (I.S.-B.); jjmoleon@ugr.es (J.J.J.-M.); mdelgado@ujaen.es (M.D.-R.); 3 Instituto de Investigación Biosanitaria ibs.GRANADA, Complejo Hospitales Universitarios de Granada/Universidad de Granada, 18071 Granada, Spain; 4 Department of Preventive Medicine and Public Health, University of Granada, 18071 Granada, Spain; 5 Division of Preventive Medicine, University of Jaén, 23071 Jaén, Spain

**Keywords:** small for gestational age, infants, maternal nutrition, physiological phenomena, Mediterranean diet, olive oil

## Abstract

To quantify the effect of a Mediterranean dietary pattern, as well as the consumption of olive oil (OO), on the risk of having a small for gestational age infants (SGA), a matched case-control study was conducted in Spain. Dietary intake during pregnancy was assessed using a validated food frequency questionnaire. Three indices were used to evaluate the adherence to Mediterranean diet (MD) (Predimed, Trichopoulou and Panagiotakos). Crude odds ratios (cOR) and adjusted odds ratios (aOR) and their 95% confidence intervals (CI) were estimated using conditional logistic regression models. Results were stratified by severity of SGA: moderate (percentiles 6–10), and severe (percentiles ≤5). For moderate, four or more points in the Predimed´s index was associated with a 41% reduction of having SGA compared with women with a score ≤3, aOR = 0.59 (95% CI 0.38–0.98); for severe, the reduction in risk was not statistically significant. Similar results were found when the other MD indexes were used. An intake of OO above 5 g/day was associated with a lower risk of SGA (aOR = 0.53, 95% CI 0.34–0.85); statistical significance was observed for moderate SGA (aOR = 0.53, 95% CI 0.30–0.96), but not for severe SGA (aOR = 0.51, 95% CI 0.24–1.07), although the magnitude of ORs were quite similar. Adherence to a MD and OO intake is associated with a reduced risk of SGA.

## 1. Introduction

A newborn’s weight is considered the main determinant of perinatal morbidity and mortality [1,2], both in the short and the long term [3,4]. The concept of small for gestational age infants (SGA) considers birth weight, gestational age, and sex of the newborn. Restricted fetal growth is associated with an increased risk of childhood morbidity and chronic diseases in adulthood such as respiratory infections, diabetes mellitus, obesity, cardiovascular diseases and psychiatric disorders [5,6].

Maternal risk factors associated with SGA can be socio-demographic variables such as age or socioeconomic status, chronic diseases, such as diabetes or hypertension, risk factors during pregnancy, including quality of prenatal care and maternal lifestyle risk factors [7]. Maternal nutrition is recognized as one of the main determinants of fetal growth. The maternal diet’s composition affects the fetal growth and determines the metabolic patterns of both mother and offspring [3,4,8,9,10].

Most studies published to date that evaluate diet during pregnancy have focused on the association between individual foods or nutrients and fetal growth [11]. The foods or nutrients associated with a lower risk of SGA are diverse depending on the study considered [12,13,14,15,16]. However, nutrition is a multidimensional exposure; nutrients or foods are not consumed in isolation, and the sources for the same nutrient can be different [17]. As such, dietary patterns may provide more useful information than an isolated nutrient or food. Among dietary patterns, the Mediterranean diet (MD) stands out for its effect on health, such that the American Dietary Guidelines recommends it because of its global source of essential nutrients and its usefulness in the prevention of diseases [18]. Furthermore, the MD can also influence the wellbeing of the mother and the fetus.

Regarding the MD and risk of SGA, the studies carried out to date are few with inconsistent results. MD is characterized by a high consumption of olive oil (OO) as the main source of fat. The Mediterranean dietary pattern and OO have been related to a reduction of intrauterine growth retardation, low birth weight and premature births [9,19]. However, there are studies, which do not include a MD pattern, that suggest that dietary patterns are not associated with the risk of SGA [20,21]. Taking into account these discrepancies, the objective of this study was to quantify the effect of the maternal MD pattern, as well as the consumption of OO, on the risk of having a SGA infant.

## 2. Materials and Methods

We used a matched case-control study. The matching criterion was the maternal age at delivery (±2 years). The study population included women who gave birth to a singleton newborn in one of five hospitals in Eastern Andalusia (Spain): The University of Jaén Hospital (UJH), Ubeda Hospital (UH), the University of Granada Hospitals (two centers) (UGH), and Poniente Hospital (PH), serving a total of 1.8 million people. Case and control groups were collected between 15 May 2012 and 15 July 2015. Ethical Approval for this study was given by the Ethics Committees of the hospitals. All women included in the study signed an informed consent.

### 2.1. Cases

Eligibility criteria for cases were: (1) Maternal residence in the hospital coverage area; (2) Birth of a single live newborn; (3) Absence of congenital malformations; and (4) SGA diagnosed according to the tables developed for the Spanish population [22]. Using neonatal growth curves (recognized by the Spanish Society of Gynecology and Obstetrics as reference for Spanish population), neonates weighting less than the 10th percentile, adjusted for gestational age at delivery and sex, were diagnosed as SGA; SGA was classified as severe if percentile was ≤5 and moderate for centiles 6–10. Nineteen women declined to participate. A total of 533 cases were selected from the four different hospitals: 79 (UJH), 46 (UH), 369 (UGH), and 39 (PH).

### 2.2. Controls

**Figure 1:**
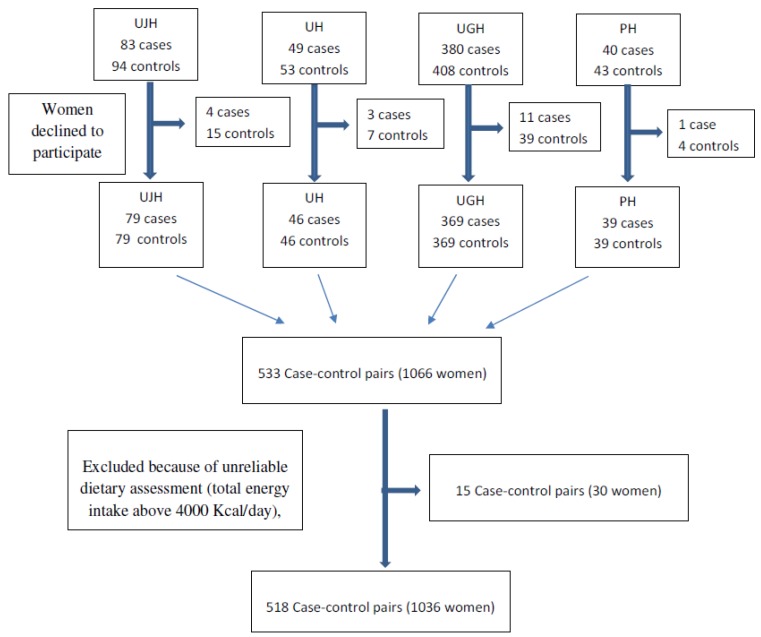
Participant flowchart. Abbreviation: The University of Jaén Hospital (UJH), Ubeda Hospital (UH), the University of Granada Hospitals (two centers) (UGH), and Poniente Hospital (PH).

A match pair by maternal age at delivery (±2 years) was selected within the week following inclusion of a case in the same hospital. Eligible women were those having a normal weight for gestational age infants with the same selection criteria used for cases (residence in the referral area of the hospital and no congenital malformations). Sixty-five women declined participation (Figure 1).

### 2.3. Data Collection

Three sources of data were used: (1) personal interviews (carried out within two days after delivery); (2) clinical charts; and (3) prenatal care records. Information on the following variables were obtained: Mother’s socio-demographic data (marital status, education level, ethnicity, socioeconomic class and occupation, monthly income, age at the beginning of the pregnancy and pre-pregnancy body mass index); obstetric history (parity and antecedent abortions, previous adverse perinatal outcomes); conditions during pregnancy (infections, preeclampsia, diabetes and other obstetric conditions); smoking during pregnancy; prescribed and over-the-counter drugs; prenatal care (number of visits and date of first visit); and birth weight (weight in grams in the delivery room). Social class were coded in five main levels (ranging from I, the highest, to V, the lowest) according to the classification of the Spanish Society of Epidemiology [23] which is close to that of the Black Report [24]. Prenatal care utilization was measured by using the Kessner index. This index includes information about both the timing of prenatal care initiation and prenatal care visits after initiation [25].

### 2.4. Dietary Assessment

The baseline questionnaire included a semi-quantitative food frequency questionnaire (FFQ) with 137 items previously translated, adapted and validated in Spanish women aged 18–74 years. Moreover, the questionnaire included open-label questions for information about the use of dietary supplements during the pregnancy [26]. The final questionnaire has been previously assessed by de la Fuente-Arillaga et al. [27]. The FFQ was based on typical portion sizes and had nine options for the frequency of intake in the previous year for each food item (ranging from never or almost never to ≥6 times/day). A dietitian updated the nutrient data bank using the information recorded in the food composition tables for Spain [28,29]. Nutrient scores were computed using ad hoc computer software specifically developed for this purpose (frequency and nutrient composition of specified portion size for each food item). After computing total energy intake, 15 matched pairs were excluded because of unreliable dietary assessment (total energy intake above 4000 kcal/day), leaving 518 pairs for analysis.

### 2.5. Mediterranean Diet Pattern Adherence Indexes

Three indices were used to evaluate the adherence to a MD:

1. Predimed, which was developed in Spain [30]. This index considers: Vegetables, legumes, fruit, fish, red and processed meat, chicken or poultry, olive oil for cooking, consumption of olive oil, butter-margarine, carbonated and/or sweetened beverages, commercial pastries, nuts and meals with sofrito (traditional sauce of tomatoes, garlic, onion, or pepper in olive oil). The total score ranged from 0 (minimum adherence) to 14 (maximum adherence). The index is configured by 12 questions on food consumption frequency and 2 questions on food intake habits considered characteristic of the Spanish Mediterranean diet. Each question was scored as 0 or 1. One point was given for using olive oil as the principal source of fat for cooking, preferring white meat over red meat, or for consuming: (1) 4 or more tablespoons (1 tablespoon = 13.5 g) of olive oil/day (including that used in frying, salads, meals eaten away from home, etc.); (2) 2 or more servings of vegetables/day; (3) 3 or more pieces of fruit/day; (4) <1 serving of red meat or sausages/day; (5) <1 serving of animal fat/day; (6) <1 cup (1 cup = 100 ml) of sugar-sweetened beverages/day; (7) 7 or more servings of red wine/week; (8) 3 or more servings of pulses/week (some seeds which can be cooked and eaten are called pulses, for example peas, beans, and lentils.); (9) 3 or more servings of fish/week; (10) fewer than 2 commercial pastries/week; (11) 3 or more servings of nuts/week; or (12) 2 or more servings/week of a dish with a traditional sauce of tomatoes, garlic, onion, or leeks sautéed in olive oil. If the condition was not met, 0 points were recorded for the category.
2. Mediterranean diet score, developed by Trichopoulou et al in Greece [31]. This index considers the following food groups: Vegetables, legumes, fruit, fish, cereals, meat, dairy products, and monounsaturated/saturated fats ratio. The median for each food group was estimated using the control group. For consumption of each typical Mediterranean food higher than the median of the consumption distribution in the control group, a person received 1 point; consumption lower received zero points. For consumption of non-Mediterranean foods lower than the median 1 point was awarded; consumption higher than the median received zero points. The total score ranged from 0 (minimum adherence to a traditional Mediterranean dietary pattern) to 8 (maximum adherence).
3. Dietary score, developed by Panagiotakos et al in Greece [32]. To estimate this index the following groups of food are considered: vegetables, legumes, fruits, fish, whole grains, potatoes, olive oil, poultry, dairy products with fat, and red meat. The total score ranged from 0 (minimum adherence) to 55 (maximum adherence), with higher values indicating higher adherence to a Mediterranean diet. Vegetables, legumes (e.g., peas, beans), fruits, fish, whole grains, and potatoes were categorized on the basis of servings/month and specifically as: 0 = never; 1 point = 1–4 servings/month; 2 points = 5–8; 3 points = 9–12; 4 points = 13–18; and 5 points = ≥18 servings/month. Consumption of red meat, poultry, and full fat dairy products (e.g., milk cheese, yogurt) was categorized as: 0 = ≥18 servings/month; 1 point = 13–17 servings/month; 2 points = 9–12; 3 points = 5–8; 4 points = 1–4; and 5 points = never. Consumption of olive oil was categorized according to the number of times it was used in a week and specifically as: 0 = never; 1 = rare; 2 = ≤1 times/weekly; 3 = 2 times/weekly; 4 = 3–6 and 5 = daily.

Alcohol consumption has been excluded in all indices. Regardless of the index used, a higher score indicates a greater adherence to MD pattern.

### 2.6. Statistical Analysis

Food and nutrient intakes were adjusted for total energy intake using the residuals method for cases and controls as it is recommended by Willet et al. [33]. Energy-adjusted food or nutrient intakes were categorized in quintiles. Crude and adjusted odds ratios (OR) and 95% CI were estimated with conditional regression logistic models. To determine the variables to be included in the multivariate analysis, the procedure described by Sun et al. [34] was followed. Intermediate variables were discarded. We ran two stepwise models, one backward and another forward, including variables with a value of *p* < 0.2 [35,36]. We constructed a list of predictors of SGA identified in other studies. Using information from stepwise models and the list of predictors, a saturated model was built, and by using a heuristic approach, variables that did not change the coefficient of the bundles by more than 10% were discarded, in order to construct a parsimonious model retaining all important confounders. Models were adjusted by income, smoking, previous preterm/LBW newborn, newborn’s gender, total energy intake, and pre-pregnancy body mass index (BMI). BMI was calculated as weight (in kg) just before pregnancy divided by height (in m) squared. Both weight and height were obtained from medical records of the women if possible, or self-reported if not. All p values are 2-tailed. Statistical significance was set at *p* < 0.05. Analyses were performed using the Stata Statistical Software version 14 (StataCorp LP, College Station, TX, USA).

## 3. Results

Table 1 shows the socio-demographic characteristics of the participant women, their lifestyles during the pregnancy and the quality of prenatal care. The educational level and monthly incomes were lower in cases than in controls. Moreover, the cases had a history of preterm deliveries or low birth weight infants more frequently compared to controls, 12.4% vs. 5.0% respectively (*p* < 0.001), and also for preeclampsia, 8.9% vs. 2.1% (*p* < 0.001). During the pregnancy, the mean weight gained per week was lower in cases than in controls, 278 g (SD 121) vs. 310 g (SD 114); *p* < 0.001.

In Table 2 the individual components of the Predimed index are analyzed. In crude analyses only item 7 (fish) was protective for the risk of SGA, whereas in adjusted analyses no item reached statistical significance; it was borderline for items 6 (legumes) and 7 (fish). The Predimed score was lower for cases than for controls, 5.1 (SD 0.07) vs. 5.4 (SD 0.06); *p* = 0.027. The relationship between the Predimed score with SGA is shown in Table 3. In crude analyses, a score of 4 and more decreased the risk, although it was not confirmed in multivariate analyses. However, SGA were stratified in two groups: moderate (percentiles 6–10) and severe (percentiles ≤5). For moderate SGA a significant association was observed with a score of ≥4 (aOR = 0.59, 95% CI, 0.38–0.98), not seen for severe SGA. No trend was observed (the higher the score the lower the risk) in any case.

Table 4 shows the results for the two Greek indexes of adherence to the Mediterranean diet, the one proposed by Trichopoulou et al. [31] and the other by Panagiotakos et al. [32] When the means of the indexes are considered, no differences between groups are observed: For Trichopoulou score, 4.2 (SD 0.07) for women with a SGA infants vs. 4.0 (SD 0.08) for the control group (*p* = 0.185); and for Panagiotakos score, 29.2 (SD 0.17) vs. 28.75 (SD 0.17) respectively (*p* = 0.083). With Trichopoulou score no trend with SGA risk was appreciated, although a score of ≥3 was associated with a lower risk of SGA (aOR 0.58, 95% CI, 0.41–0.84). This association was evident for moderate SGA (aOR = 0.49, 95% CI, 0.71–0.79), and not for severe SGA (aOR = 0.74, 95% CI, 0.41–1.33).

With Panagiotakos score, no relationship was observed when the whole group of SGAs was analyzed (Table 4). However, in the subgroup of moderate SGA, a score of ≥29 reduced the risk of SGA (aOR = 0.57, 95% CI, 0.39–0.83), not observed for severe SGA (aOR = 1.54, 95% CI, 0.98–2.48).

OO is the most characteristic component of the MD. The frequency and daily intake (grams per day) of OO and its relationship with the risk of SGA newborn is shown in Table 5. An analysis by quintiles did not reveal any association; after examining the raw data it was observed that above 10 g/day no relationship was found, therefore we used ad hoc cutoff points. No trend was detected: an intake above 10 g/day did not further decrease the risk appreciated with intakes of 5–9 g/day. This protective effect is observed in moderate SGA with a significant aOR = 0.53 (95% CI, 0.30–0.96) for an intake above 5 g/day. In severe SGA this intake did not achieve significance (aOR = 0.51, 95% CI, 0.24–1.07), being the OR figure (0.51) quite similar to that found in moderate SGA (0.53), although the sample size is smaller. These results are for all types of OO. In our study population, extra virgin OO was 79.8% of all OO intake. The analyses were repeated for extra virgin OO and no relevant differences were found with those shown in Table 5.

In Table 6, the different types of fatty acids (monounsaturated fatty acids (MUFA), polyunsaturated fatty acids (PUFA) and saturated fats) are analyzed. In our study population a 30% (standard deviation 17%) of MUFA come from OO. No significant differences were observed between cases and controls. No associations were observed either in the consumption of these fatty acids in isolation and a protective effect for SGA. No association was found with other fats (soybean oil, sunflower oil, corn oil, etc.) used for cooking and dressing meals (data not shown).

Women were asked for changes in their diet during pregnancy. A 49.7% of cases increased their intake of vegetables during pregnancy versus 47.1% of controls (*p* = 0.401). Regarding fruits both cases and controls augmented their consumption (61.0% vs. 58.6%, respectively, *p* = 0.437), and about olive oil intake no difference was also found (6.8% vs. 7.0%, *p* = 0.901).

## 4. Discussion

Our results suggest that maternal adherence to a Mediterranean dietary pattern is associated with a lower risk of SGA newborn; and mainly for those cases with a moderate degree of disease (percentiles 6–10). This relationship is consistent among the different MD indexes used. It seems that the effect depends on the diet pattern as a whole, although the role that OO alone may play is significant in itself. It must be emphasized that no dose-response was observed. According to the several indexes used after a level of intake no additional benefit was appreciated, and the same occurs with OO.

Fetal life is characterized by tremendous plasticity and the ability to respond to various environmental and lifestyle factors, including maternal nutrition [37]. In particular, maternal dietary habits can directly affect newborn weight [20]. Okubo et al. [14], in a cohort study of 803 women with dietary assessment at the beginning of pregnancy, reported that a diet characterized by a high consumption of bread, pastry, sweets and soft drinks, and a low consumption of fish and vegetables is associated with a higher risk of SGA. In contrast, Thompson et al. [16], in a case-control study (844 SGA and 870 controls) with a FFQ applied after delivery, found that a dietary pattern composed of meat, potatoes, fruits (particularly citrus fruits), green vegetables, carrots, dairy products and water is protective for SGA, aOR = 0.86 (95% CI 0.75–0.99). Similarly, other authors have found a 26%–32% reduction in the risk of SGA for dietary patterns with a higher intake of fruits, vegetables, poultry and breakfast cereals [38]. While these studies are not based on a Mediterranean population nor focus on a MD pattern, the main characteristics of these dietary patterns are very similar to the characteristics of a higher or lower compliance with a MD. Another Spanish study, based on a small sample (46 SGA and 81 controls) with assessment of diet at the third trimester of pregnancy, has also reported that a MD pattern is associated with a lower risk of SGA, although the reduction in the risk was not estimated [39].

Regarding OO intake we have found only one report from Northern Italy. In a case-control study with 555 SGA and 1966 controls Ricci et al reported that a high level of OO intake increased the risk of SGA [40] with an OR = 1.6 (95% CI, 1.0–2.5), which increased to 3.3 (95% CI, 1.4–7.8) for preterm SGA. Diet was ascertained after delivery, as in our study. It is not defined in this report what is a ‘high level’ of OO consumption. We have assessed the curve of risk between OO intake in g/day (either extra virgin or other types) and our results do not agree with theirs. Above 10 g/day the relationship between OO and SGA risk was flat: No increased risk was found for an intake above 30 g/day, nor for 50 g/day (results not shown). In fact, our results suggest that an OO intake above 10 g/day does add any further protection.

Our results regarding the severity of SGA cannot be compared with other reports because they have not defined levels of SGA below the 10th percentile. In some reports, such as that of Ricci et al. [40] SGA was classified by preterm delivery. The determinants of preterm delivery (92, 17.8%, of our cases) are different from those of SGA, and mostly not related to intrauterine growth retardation. We carried out stratified analyses by preterm delivery and not relevant association was detected (results not shown).

To date, we have not found randomized controlled clinical trials on MD and SGA [41]. Currently, the evidence available is based on observational studies and residual confounding cannot be ruled out. However, in Spain a randomized controlled clinical trial with two parallel groups has been developed with gestational diabetes as the primary outcome: the key intervention group recommendation was a daily consumption of at least 40 mL of extra virgin OO and a handful (25–30 g) of pistachios, and the same basic MD recommendations were given to the intervention and control groups [42]. Although it was not the primary outcome, a lower risk of SGA was observed for the intervention group. Nevertheless, the positive effects on cardiovascular and metabolic health with high compliance to a MD has been proven [43], and this type of diet could also have a benefit on birth weight, although evidence from trials is needed.

Our results suggest that the consumption OO of about a spoon a day (about 5 g) is enough to protect against the risk of SGA. In laboratory studies using mice, Mousavi et al. (2017) reported that a maternal diet containing extra virgin OO has positive effects on offspring birth weight, as well as better serum biochemical parameters [44]. OO is a rich source of monounsaturated fatty acids, and has been found to improve the inflammatory profile [45] as well as to lower postprandial glucose levels [46]. Furthermore, OO is a traditional component of the Spanish cuisine and used as a dressing improves the palatability of foods and facilitates an increased intake of vegetables. As previously commented, Assaf-Balut et al. (2017) showed a lower risk for SGA infants in the intervention group with the use of extra virgin OO and a handful of pistachios [42]. According to our results OO is associated with a lower risk of SGA, but not MUFA; this may suggest that other components (not type of fat) is responsible of this protective effect, such as phenols, associated with several chronic diseases [47].

In contrast, the effect of a maternal Mediterranean dietary pattern on birth weight is not observed for severe SGA (percentiles ≤5). It seems that diet may help in moderate growth retardation but not in more severe situations, where other causes cannot be counteracted by diet. It could be possible that the effect of a MD would be maintained in severe SGA but we could not observe it due to: (a) newborn weight depends on multiple factors; (b) the relevance of each factor is related to its frequency and the frequency of the other factors; (c) the presence of other causes, mainly maternal pathology related to chronic diseases or associated to pregnancy, reduces the role of MD on fetal development; (d) an interaction between dietary pattern and pathologies cannot be discarded; and (e) the study power is not enough. New studies are needed to overcome these limitations.

## 5. Strengths and Limitations

The strengths of our study are: (1) the sample is representative of a reference population of around 12,000 healthy pregnant women attending Spanish Andalusian public hospitals; (2) we used established Spanish fetal growth curves to define adequate size for gestational age [22]; (3) we employed a FFQ validated in the Spanish population [26,27]; (4) the control group was selected by density in the same hospitals as cases to avoid seasonal influences on diet recording.

We also have to recognize some limitations: (1) all questionnaires were recorded after birth but before hospital discharge, with the intention of estimating the average dietary intake during pregnancy. However, delivery and the last gestational week of pregnancy would be unlikely to change their habitual gestational dietary patterns. We assessed the change of diet during pregnancy and the differences between cases in controls were minimal (1%–2% better for cases); a differential misclassification bias cannot be rule out altogether, although given the reported data, its impact on the reported OR figures would not change appreciably; (2) the information was taken by midwives, possibly introducing a classification bias as the participating women may want to respond with answers they believe will please the midwife: a bias that would affect both groups and shift the size of the association strength toward the null value; (3) we cannot discard a memory bias, but if present, we think it would be a non-differential bias as no relationship between a particular food intake and SGA is assumed beforehand; (4) given the nature of observational studies, ours results cannot be free of residual confounding; (5) the problem of multiple comparisons: it could be possible that some associations may be appeared by chance; however, regarding the main point of the research, MD has been ascertained by three indexes and the results are consistent; and (6) the cutoff points for the MD scales and OO intake were chosen after examining the data, once that we did not appreciate any dose-response trend.

## 6. Conclusions

In conclusion, our results suggest that adherence to a MD pattern during pregnancy is associated with a reduced risk of having a baby with SGA, independently of the index used. This relationship was particularly evident in pregnant women who had moderate SGA (percentiles 6–10). Consumption of at least 5 g/day of OO is also associated with a lower risk of SGA. Bearing in mind the benefits of a Mediterranean diet on maternal, fetal and offspring health, it should be promoted before, during and after pregnancy.

## Figures and Tables

**Table 1:** Description of the study population of healthy pregnant Spanish women.

Variable	Cases (*n* = 518) *n* (%)		Controls (*n* = 518) *n* (%)		*p*-Value
Marital status					0.036
Single	37	(7.1)	42	(8.1)	
Stable couple	161	(31.1)	124	(23.9)	
Married	320	(61.8)	352	(68.0)	
Education level					0.084
Primary	112	(21.6)	93	(17.9)	
High school, not finished	42	(8.1)	28	(5.4)	
High school	185	(35.7)	190	(36.7)	
University	179	(34.6)	207	(40.0)	
Income (Euros/month)					0.009
<1000	146	(28.2)	145	(24.1)	
1000–1999	245	(47.3)	218	(42.1)	
2000–2999	99	(19.1)	129	(24.9)	
≥3000	28	(5.4)	46	(8.9)	
Kessner index (prenatal care)					0.737
Adequate	259	(50.0)	253	(48.8)	
Intermediate	185	(35.7)	182	(35.2)	
Inadequate	74	(14.3)	83	(16.0)	
Smoking during pregnancy	149	(28.8)	80	(15.4)	<0.001
Previous preterm/low birthweight Newborn	64	(12.4)	46	(5.0)	<0.001
Weight gain during pregnancy (g/week), mean (SD)	278	(121)	310	(114)	<0.001
Pre-pregnancy Body Mass Index (BMI), mean (SD)	23.1	(4.5)	23.9	(4.1)	<0.001
Total energy intake (kcal/day), mean (SD)	2547	(561)	2493	(538)	0.118
Alcohol intake (g/week), mean (SD)	4.2	(18.5)	3.1	(15.2)	0.312

SD: Standard deviation.

**Table 2:** Items of the PREDIMED score for adherence to Mediterranean diet and risk of Small for Gestational Age (SGA).

Predimed Item	Cases (*n* = 518) *n* (%)	Controls (*n* = 518) *n* (%)	cOR (95% CI)	aOR (95% CI)
Olive oil used as the main fat for cooking	502 (96.9)	508 (98.1)	0.60 (0.26–1.37)	0.73 (0.30–1.74)
Olive oil: 4+ tablespoons a day	68 (13.3)	57 (11.0)	1.22 (0.84–1.77)	1.12 (0.75–1.68)
Vegetables: 2+ servings a day	279 (53.9)	275 (53.1)	1.03 (0.81–1.81)	1.05 (0.81–1.36)
Fruit: 3+ servings a day	78 (15.1)	95 (18.3)	0.79 (0.57–1.10)	0.86 (0.61–1.22)
Red meat/sausages: <1 a day	103 (19.9)	116 (22.4)	0.85 (0.63–1.16)	0.90 (0.65–1.27)
Butter/margarine/cream: <1 serving a day	429 (82.8)	444 (85.7)	0.81 (0.58–1.13)	0.88 (0.61–1.27)
Soft drinks (sweet, carbonated): <1 a day	247 (47.7)	262 (50.6)	0.88 (0.69–1.14)	0.92 (0.70–1.21)
Legumes: 3+ servings a week	264 (51.0)	281 (54.3)	0.88 (0.69–1.12)	0.80 (0.61–1.04)
Fish: 3+ servings a week	409 (79.0)	434 (83.8)	0.72 (0.52–0.99) *	0.75 (0.53–1.07)
Cakes and pastries (not done at home): <2 units a week	141 (27.2)	170 (32.8)	0.78 (0.60–1.01)	0.88 (0.65–1.19)
Nuts: 3+ servings a week	123 (23.8)	108 (20.9)	1.20 (0.82–1.62)	1.16 (0.83–1.61)
White meat more frequently than red meat	16 (3.1)	17 (3.3)	0.94 (0.46–1.90)	1.01 (0.47–2.21)

aOR: Adjusted odds ratio by income, smoking, weight gain per week during pregnancy, previous preterm/low birthweight newborn, energy intake, and pre- pregnancy BMI. cOR: Crude odds ratio and confidence intervals (95% CI). * Significant association.

**Table 3:** Total Predimed score and risk of SGA for the entire sample and stratified on degree of SGA.

Predimed Score	Cases *n* (%)	Controls *n* (%)	cOR (95% CI)	aOR (95% CI)
**All SGA**				
	(*n* = 518)	(*n* = 518)		
≤3	86 (16.6)	58 (11.2)	1 (reference)	1 (reference)
4	85 (16.4)	87 (16.8)	0.66 (0.43–1.04)	0.81 (0.50–1.31)
5	139 (26.8)	144 (27.8)	0.67 (0.44–0.99) *	0.75 (0.49–1.15)
6	107 (20.7)	119 (23)	0.61 (0.40–0.93) *	0.67 (0.43–1.05)
>6	101 (19.5)	110 (21.2)	0.63 (0.41–0.96) *	0.77 (0.49–1.21)
*p* for trend			0.152	0.482
≤3 vs. ≥4			0.65 (0.45–0.92) *	0.74 (0.51–1.08)
**Moderate SGA, Percentiles 6–10**				
	(*n* = 323)	(*n* = 323)		
≤3	55 (17.0)	35 (10.8)	1 (reference)	1 (reference)
4	55 (17.0)	54 (16.7)	0.66 (0.37–1.18)	0.72 (0.38–1.35)
5	80 (24.8)	95 (29.4)	0.55 (0.33–0.92) *	0.51 (0.29–0.90) *
6	76 (23.5)	75 (23.2)	0.66 (0.38–1.12)	0.61 (0.34–1.09)
>6	57 (17.7)	64 (19.8)	0.59 (0.34–1.01)	0.64 (0.35–1.17)
*p* for trend			0.212	0.446
≤3 vs. ≥4			0.60 (0.38–0.94) *	0.59 (0.38–0.98) *
**Severe SGA, Percentiles ≤5**				
	(*n* = 195)	(*n* = 195)		
≤3	31 (15.9)	23 (11.8)	1 (reference)	1 (reference)
4	30 (15.4)	33 (16.9)	0.70 (0.34–1.42)	0.93 (0.43–2.02)
5	59 (30.3)	49 (25.1)	0.97 (0.50–1.87)	1.21 (0.59–2.50)
6	31 (15.9)	44 (22.6)	0.53 (0.26–1.06)	0.64 (0.31–1.35)
>6	44 (22.6)	46 (23.6)	0.70 (0.36–1.37)	0.92 (0.44–1.95)
*p* for trend			0.810	0.461
≤3 vs. ≥4			0.72 (0.41–1.27)	0.91 (0.49–1.68)

aOR: Adjusted odds ratio by income, smoking, previous preterm/low birthweight newborn, energy intake, newborn’s gender, and pre-pregnancy BMI. cOR: Crude odds ratio and confidence intervals (95% CI). * Significant association.

**Table 4:** Trichopoulou and Panagiotakos scores and risk of SGA for entire sample and stratified on degree of SGA.

	Cases *n* (%)	Controls *n* (%)	cOR (95% CI)	aOR (95% CI)
**Trichopoulou Score**				
**All SGA**				
	(*n* = 518)	(*n* = 518)		
≤2	109 (21.0)	74 (14.3)	1 (reference)	1 (reference)
3–4	203 (39.2)	228 (44.0)	0.59 (0.41–0.85) *	0.53 (0.36–0.78) *
5–6	161 (31.1)	180 (34.8)	0.59 (0.40–0.86) *	0.59 (0.39–0.88) *
>6	45 (8.7)	36 (7.0)	0.84 (0.50–1.43)	0.92 (0.51–1.64)
*p* for trend			0.289	0.612
≤2 vs. ≥3			0.62 (0.42–0.86) *	0.58 (0.41–0.84) *
**Moderate SGA, Percentiles 6–10**				
	(*n* = 323)	(*n* = 323)		
≤2	71 (22.0)	43 (13.3)	1 (reference)	1 (reference)
3–4	135 (41.8)	143 (44.3)	0.52 (0.33–0.82) *	0.55 (0.34–0.89) *
5–6	95 (29.4)	116 (35.9)	0.43 (0.27–0.69) *	0.47 (0.28–0.78) *
>6	22 (6.8)	21 (6.5)	0.73 (0.37–1.46)	0.73 (0.35–1.52)
*p* for trend			0.040	0.075
≤2 vs. ≥3			0.53 (0.34–0.82) *	0.49 (0.31–0.79) *
**Severe SGA, Percentiles ≤5**				
	(*n* = 195)	(*n* = 195)		
≤ 2	38 (19.4)	31 (15.9)	1 (reference)	1 (reference)
3–4	68 (34.9)	85 (43.6)	0.62 (0.34–1.12)	0.57 (0.29–1.10)
5–6	66 (33.9)	64 (32.8)	0.84 (0.46–1.54)	0.77 (0.40–1.51)
>6	23 (11.8)	15 (7.7)	1.23 (0.57–2.68)	1.44 (0.60–3.43)
*p* for trend			0.437	0.302
≤2 vs. ≥3			0.77 (0.45–1.32)	0.74 (0.41–1.33)
**Panagiotakos Score**				
**All SGA**				
	(*n* = 518)	(*n* = 518)		
≤ 26	129 (24.9)	116 (22.4)	1 (reference)	1 (reference)
27–28	107 (20.7)	103 (19.9)	0.93 (0.63–1.35)	0.93 (0.62–1.45)
29–30	109 (21.0)	120 (23.2)	0.80 (0.56–1.16)	0.77 (0.51–1.15)
31–32	95 (18.3)	77 (14.9)	1.09 (0.73–1.63)	1.05 (0.67–1.65)
> 32	78 (15.1)	102 (19.7)	0.68 (0.46–1.02)	0.67 (0.43–1.05)
*p* for trend			0.078	0.097
≤28 vs. ≥29			0.87 (0.68–1.12)	0.84 (0.64–1.11)
**Moderate SGA, Percentiles 6–10**				
	(*n* = 323)	(*n* = 323)		
≤26	91 (28.2)	69 (21.4)	1 (reference)	1 (reference)
27–28	71 (22.0)	58 (18.0)	0.91 (0.56–1.48)	1.00 (0.58–1.22)
29–30	59 (18.3)	83 (25.7)	0.52 (0.32–0.84) *	0.49 (0.29–0.85) *
31–32	56 (17.3)	45 (13.9)	0.90 (0.53–1.52)	0.85 (0.46–1.57)
>32	46 (14.2)	68 (21.0)	0.48 (0.28–0.80) *	0.47 (0.26–0.86) *
*p* for trend			0.008 *	0.015 *
≤28 vs. ≥29			0.62 (0.45–0.87) *	0.57 (0.39–0.83) *
**Severe SGA, Percentiles ≤5**				
	(*n* = 195)	(*n* = 195)		
≤26	38 (19.5)	47 (24.1)	1 (reference)	1 (reference)
27–28	36 (18.5)	45 (23.1)	1.00 (0.54–1.87)	0.79 (0.39–1.59)
29–30	50 (25.6)	37 (19.0)	1.63 (0.89–2.98)	1.53 (0.79–2.97)
31–32	39 (20.0)	32 (16.4)	1.52 (0.80–2.90)	1.42 (0.69–2.90)
>32	32 (16.4)	34 (17.4)	1.22 (0.64–2.33)	1.06 (0.51–2.23)
*p* for trend			0.610	0.770
≤28 vs. ≥29			1.33 (0.81–2.20)	1.19 (0.69–2.07)

aOR: Adjusted odds ratio by income, smoking, previous preterm/low birthweight newborn, energy intake, newborn’s gender, and pre-pregnancy BMI; cOR: Crude odds ratio and confidence intervals (95% CI); * Significant association.

**Table 5:** Daily intake (g/day) of olive oil (OO) and risk of SGA for entire sample and stratified on degree of SGA.

OO Daily Intake (g/day)	Cases *n* (%)	Controls *n* (%)	cOR (95% CI)	aOR (95% CI)
**All SGA**				
<5	71 (13.7)	42 (8.1)	1 (reference)	1 (reference)
5–9.9	60 (11.6)	70 (13.5)	0.48 (0.28–0.82) *	0.52 (0.29–0.93) *
10–19.9	150 (29.0)	159 (30.7)	0.53 (0.34–0.85) *	0.55 (0.33–0.89) *
20–29.9	149 (28.8)	170 (32.8)	0.49 (0.31–0.78) *	0.49 (0.30–0.81) *
≥30	88 (17.0)	77 (14.9)	0.64 (0.39–1.06)	0.61 (0.51–1.05)
*p* for trend			0.735	0.961
<5 vs. ≥5			0.53 (0.35–0.81) *	0.53 (0.34–0.85) *
**Moderate SGA Percentiles 6–10**				
<5	45 (13.9)	26 (8.1)	1 (reference)	1 (reference)
5–9.9	42 (13.0)	47 (14.5)	0.48 (0.25–0.94) *	0.55 (0.26–1.16)
10–19.9	103 (31.9)	95 (29.4)	0.60 (0.34–1.07)	0.59 (0.31–1.12)
20–29.9	85 (26.3)	114 (35.0)	0.41 (0.23–0.74) *	0.41 (0.21–0.78) *
≥30	48 (14.9)	42 (13.0)	0.64 (0.34–1.22)	0.62 (0.31–1.25)
*p* for trend			0.964	0.868
<5 vs. ≥5			0.53 (0.31–0.89) *	0.53 (0.30–0.96) *
**Severe SGA, Percentiles ≤5**				
<5	26 (13.3)	16 (8.2)	1 (reference)	1 (reference)
5–9.9	18 (9.2)	23 (11.8)	0.47 (0.19–1.16)	0.46 (0.17–1.22)
10–19.9	47 (24.1)	64 (32.8)	0.44 (0.20–0.94) *	0.44 (0.19–0.99) *
20–29.9	64 (32.8)	57 (29.2)	0.68 (0.32–1.46)	0.61 (0.26–1.34)
≥30	40 (20.5)	35 (18.0)	0.66 (0.29–1.50)	0.59 (0.24–1.44)
*p* for trend			0.568	0.853
<5 vs. ≥5			0.55 (0.27–1.10)	0.51 (0.24–1.07)

aOR: Adjusted odds ratio by income, smoking, previous preterm/low birthweight newborn, energy intake, newborn’s gender, and pre-pregnancy BMI; cOR: Crude odds ratio and confidence intervals (95% CI); * Significant association.

**Table 6:** Type of fatty acids in quintiles (g/day) and risk of SGA.

	Cases *n* (%)	Controls *n* (%)	cOR (95% CI)	aOR (95% CI)
**MUFA**				
Q1 (≤37.96 g/day)	99 (19.1)	104 (20.1)	1 (reference)	1 (reference)
Q2 (37.97–43.09)	102 (19.7)	104 (20.1)	1.04 (0.70–1.53)	1.11 (0.73–1.69)
Q3 (43.10–47.18)	99 (19.1)	103 (19.9)	1.01 (0.69–1.49)	1.14 (0.75–1.74)
Q4 (47.19–53.98)	110 (21.2)	104 (20.1)	1.11 (0.49–1.66)	1.12 (0.72–1.73)
Q5 (>53.98)	108 (20.9)	103 (19.9)	1.10 (0.66–1.61)	1.03 (0.69–1.55)
*p* for trend			0.710	0.731
**PUFA**				
Q1 (≤13.32 g/day)	91 (17.6)	104 (20.1)	1 (reference)	1 (reference)
Q2 (13.33–15.39)	88 (17.0)	104 (20.1)	0.98 (0.65–1.49)	1.03 (0.66–1.60)
Q3 (15.40–17.30)	106 (20.5)	103 (19.9)	1.19 (0.81–1.75)	1.20 (0.79–1.82)
Q4 (17.31–20.12)	116 (21.2)	104 (20.1)	1.31 (0.87–1.96)	1.25 (0.81–1.95)
Q5 (>20.12)	117 (22.6)	103 (19.9)	1.34 (0.88–2.01)	1.19 (0.77–1.84)
*p* for trend			0.269	0.736
**Saturated**				
Q1 (≤29.90 g/day)	107 (20.7)	104 (20.1)	1 (reference)	1 (reference)
Q2 (29.91–33.80)	99 (19.1)	104 (20.1)	0.91 (0.62–1.35)	0.92 (0.61–1.39)
Q3 (33.81–36.91)	92 (17.8)	103 (19.9)	0.87 (0.59–1.29)	0.91 (0.59–1.41)
Q4 (36.92–40.79)	93 (18.0)	104 (20.1)	0.88 (0.59–1.31)	0.81 (0.53–1.25)
Q5 (>40.79)	127 (24.5)	103 (19.9)	1.21 (0.82–1.79)	1.05 (0.69–1.59)
*p* for trend			0.067	0.372

aOR: Adjusted odds ratio by income, smoking, previous preterm/low birthweight newborn, energy intake, newborn’s gender, and pre-pregnancy BMI; cOR: Crude odds ratio and confidence intervals (95% CI); MUFA: Monounsaturated fatty acids; PUFA: Polyunsaturated fatty acids.
